# Cell Cycle-Related Gene SPC24: A Novel Potential Diagnostic and Prognostic Biomarker for Laryngeal Squamous Cell Cancer

**DOI:** 10.1155/2023/1733100

**Published:** 2023-01-21

**Authors:** Jialei Chen, Jing Luo, Jing He, Xianyao Jiang, Ning Jiang, Changhong Yang, Shixun Zhong

**Affiliations:** ^1^Department of Otolaryngology, The First Affiliated Hospital of Chongqing Medical University, No. 1 Youyi Road, Yuzhong District, Chongqing 400016, China; ^2^Department of Pathology and Pathophysiology, Chongqing Medical University, Chongqing, China; ^3^Molecular Medicine and Cancer Research Center, College of Basic Medical Sciences, Chongqing Medical University, Chongqing 400016, China; ^4^Department of Pathology, Chongqing Medical University, Chongqing, China; ^5^Department of Pathology, The First Affiliated Hospital of Chongqing Medical University, Chongqing, China; ^6^Department of Bioinformatics, College of Basic Medicine, Chongqing Medical University, Chongqing 400016, China

## Abstract

Laryngeal squamous cell cancer (LSCC) is a common malignant tumor with a high degree of malignancy, and its etiology remains unclear. Therefore, screening potential biomarkers is necessary to facilitate the treatment and diagnosis of LSCC. Robust rank aggregation (RRA) analysis was used to integrate two gene expression datasets of LSCC patients from the Gene Expression Omnibus (GEO) database and identify differentially expressed genes (DEGs) between LSCC and nonneoplastic tissues. A gene coexpression network was constructed using weighted gene correlation network analysis (WGCNA) to explore potential associations between the module genes and clinical features of LSCC. Combining differential gene expression analysis and survival analysis, we screened potential hub genes, including CDK1, SPC24, HOXB7, and SELENBP1. Subsequently, western blotting and immunohistochemistry were used to test the protein levels in clinical specimens to verify our findings. Finally, four candidate diagnostic and prognostic biomarkers (CDK1, SPC24, HOXB7, and SELENBP1) were identified. We propose, for the first time, that SPC24 is a gene that may associate with LSCC malignancy and is a novel therapeutic target. These findings may provide important mechanistic insight of LSCC.

## 1. Introduction

Laryngeal squamous cell carcinoma (LSCC) is an extremely common malignant tumor of the head and neck and is typically characterized by squamous cell carcinoma [1]. LSCC has the second highest mortality among malignant tumors of the respiratory system, and its etiology thus far remains unclear [2]. Epidemiological data have confirmed that LSCC is related to smoking, drinking, virus infection, and other factors and often results from the synergistic effects of numerous carcinogenic factors [3]. Recently, the incidence of LSCC has obviously risen, with higher rates in males than females (5.8 cases/100,000 vs. 1.2 cases/100,000, respectively) [4]. Moreover, LSCC is highly malignant and aggressive and has a dismal prognosis and a high rate of relapse [5]. LSCC is locally invasive and metastatic, and the current clinical strategies mainly adopt multidisciplinary comprehensive treatments based on surgery, which preserves and adapts the function of the larynx to the greatest extent possible while completely eradicating the tumor [6]. However, most patients are at advanced stages (III and IV) at the time of their initial diagnosis [7]. These patients need both radical resection and radiotherapy, and chemotherapy with or without biological agents is recommended. The former may lead to cosmetic deformities and functional impairment, which will seriously affect their daily lives [8]. At present, molecular testing does not influence the selection of treatments for LSCC, and surgical resection is the only feasible method for complete eradication; however, the surgical quality is not easily controlled [4]. Therefore, new therapeutic targets and biomarkers are urgently needed for diagnostic and prognostic predictions in LSCC.

Many studies have identified some effective biomarkers that can be used as prognostic markers for patients with LSCC. Kim et al. [9] have demonstrated that FGFR1 amplification could be used as a diagnostic marker for predicting disease-free survival in patients with LSCC. Zhou et al. [10] suggested that the expression of BCL11A was upregulated in some LSCC tissue, and its upregulation was associated to the lymph node metastasis and survival of patients. Ogino et al. [11] showed that downregulation of class I human leukocyte antigens (HLAs) shortened the survival times of LSCC patients and could serve as an independent prognostic biomarker. However, they face the problems of single origin of tumor tissue and small sample size. There are only a few studies on the pathogenesis of LSCC, and the results are not satisfactory.

In recent years, the use of bioinformatics methods to explore and analyze multiple disease-related genes or proteins has become increasingly effective. Numerous gene expression datasets of LSCC patients in public databases, such as the Gene Expression Omnibus (GEO, http://igc1.salk.edu:3838/bart/) and The Cancer Genome Atlas (TCGA, https://cancergenome.nih.gov/), can be downloaded for free. These datasets contain a substantial amount of valuable information that can be reused based on new algorithms and analysis methods. Therefore, the aim of this study was to integrate data from GEO and TCGA through systematic bioinformatics tools in order to screen hub gene that are closely associated to the occurrence and progression of LSCC. Subsequently, we herein identified four hub genes, CDK1, SPC24, HOXB7, and SELEBNP1, as diagnostic and prognostic biomarkers of LSCC.

## 2. Materials and Methods

### 2.1. Research Plan

**Figure 1:**
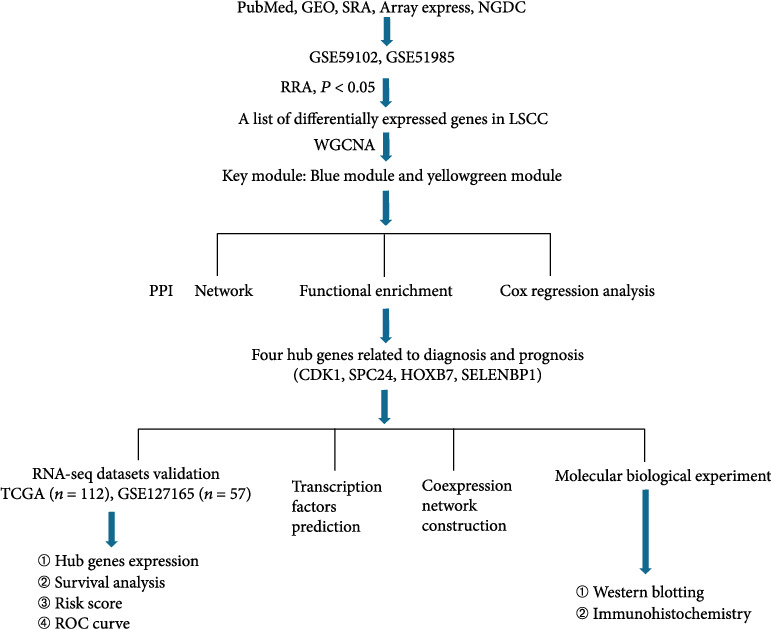
Diagram illustrating the study's design process.

Hub genes were identified, functionally analyzed, and validated according to the flow chart shown in Figure 1.

### 2.2. Gene Expression Dataset Collection

All LSCC-related datasets were downloaded from GEO, a public gene expression and hybridization array data repository [12]. Each dataset was then comprehensively evaluated according to specific criteria. The inclusion criteria were as follows: (1) the genome expression profiles were compared between LSCC samples and adjacent nonneoplastic samples through gene microarray technology, and raw data were provided, and (2) the DEGs between LSCC and adjacent nonneoplastic samples were detected in human tumor tissue and not in human cell lines or whole blood. GSE59102 [13] and GSE51985 [14] were selected as the datasets for subsequent analysis. An overview of the two GEO datasets included in this study can be found in Table 1. Finally, 39 LSCC samples and 23 adjacent nonneoplastic samples were involved in the study.

### 2.3. Identification of DEGs

Robust rank aggregation (RRA) analysis uses probabilistic models to integrate gene ranks and assign significance scores to each gene, which enables the integration of gene expression profiles from multiple datasets [15]. The DEGs between LSCC samples and adjacent nonneoplastic samples were analyzed by the Bioinformatics Array Research Tool (BART, http://igc1.salk.edu:3838/bart/) [16], and RRA analysis was used to integrate the DEGs of the two datasets. The *P* value of each gene was calculated, and the Bonferroni correction was used to reduce the number of false-positive. DEGs with a *P* value < 0.05 and a |log*FC*| > 1.0 were considered statistically significant.

### 2.4. Construction of the Coexpression Network

In this study, we performed WGCNA software to construct the DEG coexpression network. In the first step, we calculated Pearson's correlation coefficients of the DEGs using a linear correlation function [17]. Two genes were correlated if the absolute value of the correlation coefficient was greater than or equal to 0.8. In the second step, we used a dynamic tree cutting algorithm to construct a cluster tree for the gene coexpression network, and the minimum number of genes per gene module was set to 30. In the third step, the correlations and confidence levels of each gene in all module were analyzed to determine the phenotypic traits. The most relevant and significant modules were selected as core modules for subsequent analysis.

### 2.5. Functional Enrichment Analysis of Merged Module Genes

The DAVID database (https://david.ncifcrf.gov/) [18] was used to conduct Gene Ontology (GO) [19] and Kyoto Encyclopedia of Genes and Genomes (KEGG) [20] pathway enrichment analyses of the merged key module genes. The Benjamini and Hochberg test was performed, and *P* values < 0.05 indicated statistical significance.

### 2.6. PPI Network

First, we used the Search Tool for the Retrieval of Interacting Genes database (STRING, http://www.string-db.org/) to construct a PPI network for the merged key modules [21]. We then imported our results into Cytoscape software for visualization and utilized the MCODE plug-in to search for clustered subnetworks [22]. Next, we set the following default parameters: node score cutoff = 0.2, k − core = 2, degree cutoff = 5, and maximum depth = 100.

### 2.7. Identification of Prognosis-Related Genes

RNA sequencing data associated to the survival of 112 LSCC patients were downloaded from TCGA. The clinicopathological parameters of the LSCC patients, including their age, sex, pathological stage, living status, smoking status, and overall survival (OS) status, were used for further analysis to identify hub genes associated with diagnosis and prognosis. Statistical significance was set at *P* < 0.05. A univariate Cox proportional hazards regression model was applied to screen candidate genes that were closely related to survival according to *P* < 0.05, and a multivariate Cox proportional hazards regression model was established to identify gene markers correlated with prognosis based on a cutoff of *P* < 0.05. Patients with LSCC were classified into a low-risk cohort and a high-risk cohort according to their median prognostic risk score, and the Kaplan-Meier curve analysis revealed the OS rates for the low-risk and high-risk cohorts. The accuracies of the prognostic gene markers to predict the 5-year survival of patients with LSCC were determined by receiver operating characteristic (ROC) curve analysis, as the area under the ROC curve (AUC) can assess the predictive performance.

### 2.8. Prediction of Transcription Factors and Construction of a Coexpression Network of Hub Genes

The Human Transcription Factor Database (HumanTFDB) functions to screen, classify, and annotate human transcription factor genes. Transcription factors in the identified hub genes were predicted by the HumanTFDB, and a coexpression network was constructed using STRING to analyze the interactions between the hub genes.

### 2.9. Ethics Statement

All patients and their families signed informed consent forms and agreed to provide laryngeal tissue for scientific research. This research was approved by the Ethics Committee of the First Affiliated Hospital of Chongqing Medical University (Chongqing, China).

### 2.10. Collection of Laryngeal Tissue

The laryngeal tissues in this study were acquired from patients who were diagnosed with LSCC before and after surgery who underwent total laryngectomy at the Department of Otolaryngology, First Affiliated Hospital of Chongqing Medical University.

### 2.11. Verification of Differential Gene Expression

To validate the differential expression of genes in human LSCC and normal tissues, RNA sequencing datasets were collected from PubMed, GEO, SRA, ArrayExpress, and the National Genomics Data Center (NGDC), and only GSE127165 contained our screen hub genes as determined by bioinformatics analysis. GSE127165 (SRP186837, PMID: 32487167) includes 57 LSCC tissues and 57 adjacent nonneoplastic tissues [23].

### 2.12. Verification of Differential Protein Expression

Western blot (WB) was used to analyze the differences in protein expression between LSCC tissues and adjacent nonneoplastic tissues. After grinding the tissues on ice, the proteins were cleaved by RIPA buffer and PMSF protease inhibitor. The protein homogenate was then cryogenically centrifuged at 12000 rpm, and the supernatant was carefully collected. The target proteins were separated by sodium dodecyl sulfate polyacrylamide gel electrophoresis (SDS-PAGE), transferred to a polyvinylidene fluoride (PVDF) membrane, and then incubated at 37°C for 2 hours with a blocking solution. The membranes were then sequentially incubated with the corresponding specific primary antibodies overnight at 4°C and with the appropriate secondary antibody for 2 hours. The target proteins were detected by enhanced diaminobenzidine (DAB) solution, and ImageJ was used to analyze the gray-scale values. The following antibodies were used in this experiment: anti-HOXB7 (Proteintech, 12612-1-AP, 1 : 500), anti-SPC24 (Novus, NBP2-47264, 1 : 500), anti-SELENBP1 (Novus, NBP1-54805, 1 : 500), anti-CDK1 (Abcam, ab133327, 1 : 500), and horseradish peroxidase- (HRP-) conjugated anti-rabbit IgG (H+L) (ABclonal, AS014, 1 : 2000).

Immunohistochemistry (IHC) assays were performed to assess the protein levels in LSCC samples and adjacent nonneoplastic samples. Paraffin-embedded sections of LSCC tissues and adjacent nonneoplastic tissues were sliced to a thickness of 3 *μ*m and then subsequently dewaxed with xylene and ethanol and heated in antigen recovery solution (EDTA). Endogenous peroxidase activity was blocked with normal goat serum. After preliminary treatment, the sections were incubated overnight at 4°C with the primary antibody and then treated with an HRP-conjugated secondary antibody. Positive reactions were revealed with DAB solution, and the samples were counterstained with hematoxylin. After dehydration and xylene treatment, the sections were sealed with neutral resin and observed and photographed under a microscope. The primary antibodies were as follows: anti-HOXB7 (Novus, NBP2-14098, 1 : 50), anti-SPC24 (Novus, NBP2-47264, 1 : 50), anti-SELENBP1 (Novus, NBP1-54805, 1 : 50), and anti-CDK1 (Abcam, ab133327, 1 : 50).

### 2.13. Statistical Analysis

Statistical analyses were performed using R (version 3.6.0), GraphPad Prism 9.0 (GraphPad, San Diego, USA), or SPSS 20.0. The following R software packages were used for processing: survival, ggplot2, RRA, and WGCNA.

## 3. Results

### 3.1. Identification of DEGs

**Figure 2:**
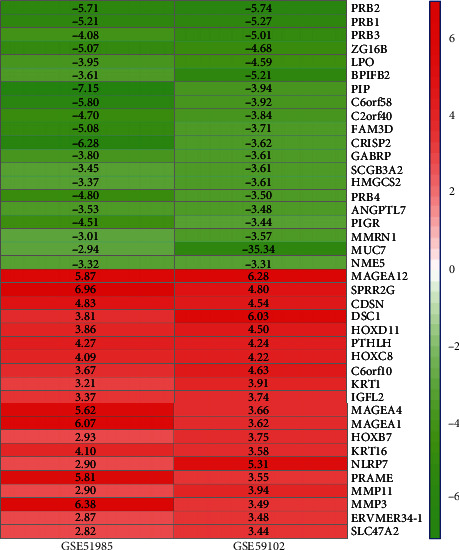
RRA analysis identifies robust DEGs. *P* value-based heatmap showing top 20 genes upregulated and downregulated. The rows represent genes and the columns represent datasets. Red indicates upregulation; green indicates downregulation.

A total of 1622 upregulated genes and 1634 downregulated genes were identified by RRA analysis. The top 20 upregulated and downregulated genes are shown in the heatmap (Figure 2).

### 3.2. Network Construction via WGCNA

**Figure 3:**
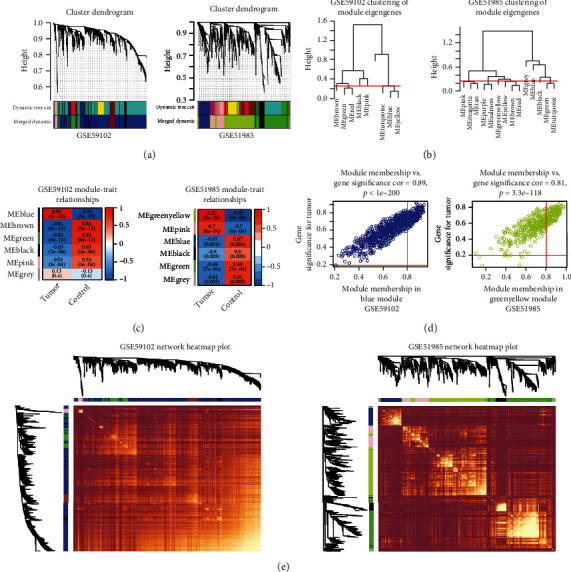
The construction of coexpression modules in R using WGCNA. (a) Module clustering dendrogram of GSE59102 and GSE51985. (b) Clustering of module eigengenes in GSE59102 and GSE51985. Under the red line was the merging threshold, and groups of eigengenes below it represented modules whose expression profiles should be merged. (c) Correlation between key modules and clinical features of GSE59102 and GSE51985. Blue module in GES59102 and green-yellow module in GSE51985 were most strongly correlated with tumor. (d) The scatterplot depicts the GS vs. MM in the blue module in GSE59102 and the GS vs. MM in green-yellow module in GSE51985. (e) Coexpression clustering heatmap of GSE59102 and GSE51985. The different colors of the horizontal axis and vertical axis represented different modules. The yellow area in the center of the image represented the connectivity of the different modules.

There were no obvious outliers in the GSE59102 and GSE51985 datasets after sample clustering. The R package WGCNA was used to build a weighted coexpression network. In GSE59102, the power *β* was set to the soft threshold parameter of *β* = 7 (scale-free *R*^2^ = 0.88) to ensure a scale-free network. In GSE51985, the power *β* was set to the soft threshold parameter of *β* = 10 (scale-free *R*^2^ = 0.85) to ensure that the network was scale-free. Nine modules were found in GSE59102, and 14 modules were found in GSE51985 (Figure 3). The MEDissThres parameter was set to 0.25 to merge the closed modules into new modules with a feature vector difference of <0.25 (Figure 3). After the merger, 6 modules were obtained from GSE5102, and 7 modules were obtained from GSE51985. In GSE59102, the black module included 70 genes, the green module included 164 genes, the pink module included 38 genes, the blue module included 927 genes, the brown module included 116 genes, and the gray module included 1 gene. In GSE51985, the black module included 98 genes, the blue module included 155 genes, the gray module included 26 genes, the pink module included 163 genes, the green-yellow module included 503 genes, and the green module included 371 genes. Because the genes in the gray modules from both two datasets did not belong to any functional module, analysis was not needed. In addition, the correlations between the gene expression profile and each trait and between the gene expression profile and each module eigengene were identified by analyzing the gene significance (GS) and module membership (MM) scores. The results indicated that the blue module (0.92, *P* = 5*e* − 18) in GSE59102 and the green-yellow module (0.82, *P* = 9*e* − 06) in GSE59102 were most strongly correlated with tumors (Figure 3). Moreover, genes belonging to the blue and green-yellow modules had the highest positive correlation with LSCC (Figure 3). The selected genes are shown in the heatmap (Figure 3).

### 3.3. Functional Enrichment Analysis of the Merged Module Genes

**Figure 4:**
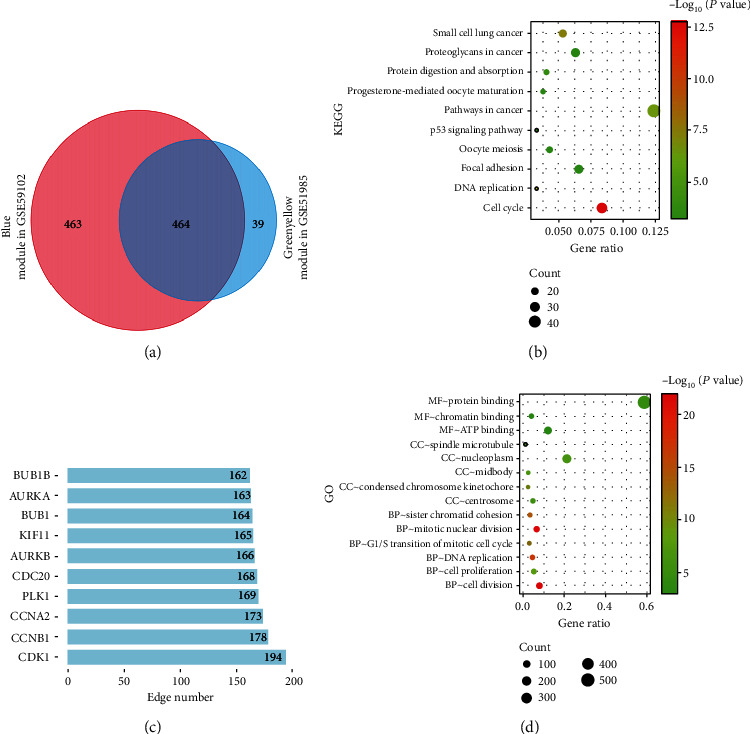
PPI network analysis and functional enrichment analysis. (a) Merged 2 key module genes. (b) Kyoto Encyclopedia of Genes and Genomes enrichment analysis. The abscissa represented GeneRatio, and the ordinate represented the item name. (c) Edge number of each key gene in PPI network. Gene names were displayed along the *y*-axis, and the number of genes adjacent to them was displayed along the *x*-axis (d) The enrichment results of biological process, cellular component, and molecular function.

A total of 966 genes were obtained by merging the blue module genes of GSE59102 and the green-yellow module genes of GSE51985. Among them, 464 genes overlapped, 463 genes belonged to only the blue module, and 39 genes belonged to only the green-yellow module (Figure 4). GO enrichment analysis showed that the merged genes were mainly enriched in cell division, mitotic nuclear division, and protein binding. KEGG enrichment analysis indicated that the merged genes were mostly enriched in the cell cycle and cancer pathways (Figure 4).

### 3.4. PPI Network

The merged genes (966 genes) were imported into the STRING database to construct a PPI network consisting of 863 nodes and 12081 edges. STRING software was used to identify 10 significant proteins, among which CDK1 was identified as the most valuable protein connecting 194 nodes (Figure 4). Cluster subnets were generated by using the MCODE plug-in, and a total of 29 clusters were generated. Among them, cluster 1 had the highest score (MCODE score = 104), with 122 nodes and 6298 edges. Cluster 2 had the second highest score (MCODE score = 13), with 30 nodes and 201 edges, and cluster 3 had the third highest score (MCODE score = 13), with 26 nodes and 172 edges. Functional enrichment analysis of the top 3 clusters was also conducted. The above results suggest that the genes in these 3 clusters are related to LSCC (Figure S1).

### 3.5. Identification of Prognosis-Related Genes

**Figure 5:**
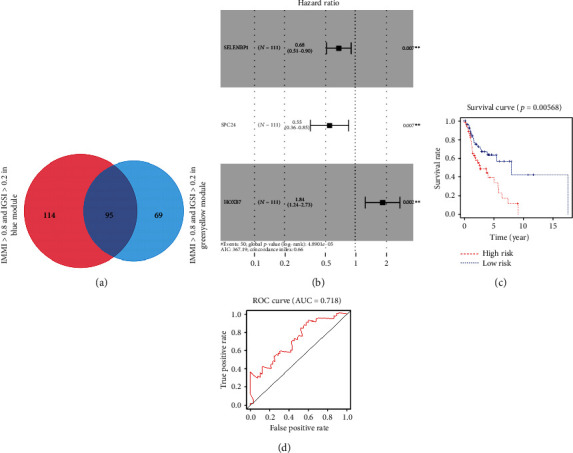
Survival prognosis model of the hub genes. (a) 278 genes were obtained after merging blue module and green-yellow module. (b) Multivariate Cox proportional hazards regression analysis identified 3 hub genes. (c) Survival analysis indicated that the OS rate of low-risk patients was much better than that of high-risk patients in the TCGA cohort. (d) To determine the most optimal cutoff value for dividing LSCC patients into high-risk and low-risk groups, ROC analysis was performed.

**Figure 6:**
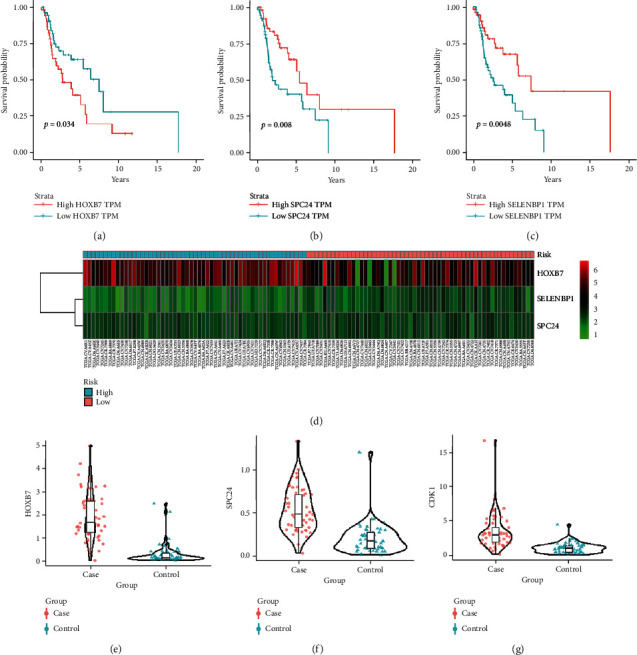
Validation of hub gene. The correlation between the expression levels of hub genes and the survival of LSCC patients. (a) HOXB7, (b) SPC24, and (c) SELENBP1. (d) Heatmap showing the changing process between down- and upregulation of 3 hub genes. Each column represents a sample and each row represents a gene. The validation of hub gene expression in GSE127165. (e) HOXB7, (f) SPC24, and (g) CDK1.

Based on the cutoff criteria (|*MM*| > 0.8 and |*GS*| > 0.2), 209 genes from the blue module and 164 genes from the green-yellow module were screened. The two gene sets were merged to obtain 278 genes (Figure 5). The univariate Cox proportional hazards regression analysis was performed on the merged gene sets using TCGA data. Based on the cutoff criterion of *P* < 0.05 as the standard, 11 genes were identified, including HOXC13, CCDC86, ERVMER34-1, SELENBP1, PNPLA7, SPC24, PKMYT1, HOXB7, MB21D2, TFRC, and IGF2BP3. The above genes were further analyzed by the multivariate Cox proportional hazards regression. According to the criterion of *P* < 0.05, HOXB7, SELENBP1, and SPC24 were identified as hub genes (Figure 5). SPC24 was identified as an independent prognostic factor for LSCC patients and was significantly correlated with their survival status in the TCGA data (Tables 2 and 3). The results for the remaining genes are shown in Tables S1–S6. With the prognostic gene signature, LSCC patients were divided into low- and high-risk cohorts based on the median prognostic risk score. The Kaplan-Meier survival analysis showed that the OS rate of low-risk patients was much better than that of high-risk patients in the TCGA cohort (Figure 5). Based on the ROC analysis, we determined the optimal cutoff value (0.678), at which the specificity and sensitivity were 0.546 and 0.861, respectively. The prognostic gene markers accurately predicted survival because the 5-year survival rate had an AUC value of 0.775 (Figure 5).  Figure 6 shows the correlation between the hub gene expression levels and LSCC patient survival and the distributions of the expression levels of the 3 hub genes in the low-risk and high-risk groups. The expression of CDK1 had no significant effect on OS. The expression levels of SPC24 (adj. *P* value = 6.16*E*-24), HOXB7 (adj. *P* value = 2.19*E*-62), and CDK1 (adj. *P* value = 4.46*E*-24) were higher in LSCC tissues than in normal tissues in the GSE127165 dataset, but no expression data were available for SELENBP1.

### 3.6. Prediction of Transcription Factors and Coexpression Network Construction

**Figure 7:**
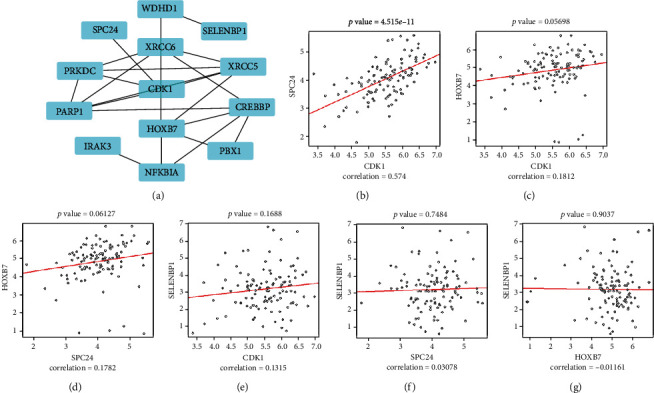
Coexpression network of four hub genes. (a) Visualized coexpression of four hub genes. Visualization of correlation analysis of gene expression values of the four hub genes. (b) SPC24 vs. CDK1. (c) HOXB7 vs. CDK1. (d) HOXB7 vs. SPC24. (e) SELENBP1 vs. CDK1. (f) SELENBP1 vs. SPC24. (g) SELENBP1 vs. HOXB7. *P* < 0.05 considered that there is a significant correlation between the expression of the two genes.

In the HumanTFDB database, the CDK1 mRNA expression values in TCGA cancers range from 0 RSEM to 2000 RSEM, while the HOXB7 mRNA expression values range from 0 RSEM to 1061.6 RSEM. The HumanTFDB database predicted CDK1 and HOXB7 to transcription factors based on their mRNA expression values in head and neck squamous cell carcinoma (HNSC) of 882.7 RSEM and 4.9 RSEM, respectively. Coexpression networks based on four hub genes were constructed in STRING. The results showed that CDK1 and SPC24 were coexpressed, which was confirmed by correlation analyses of the expression values of the four hub genes (Figure 7). Functional enrichment analysis indicated that CDK1 and spc24 coexpressions mainly function in the cell cycle pathway.

### 3.7. Verification of Differential Protein Expression

**Figure 8:**
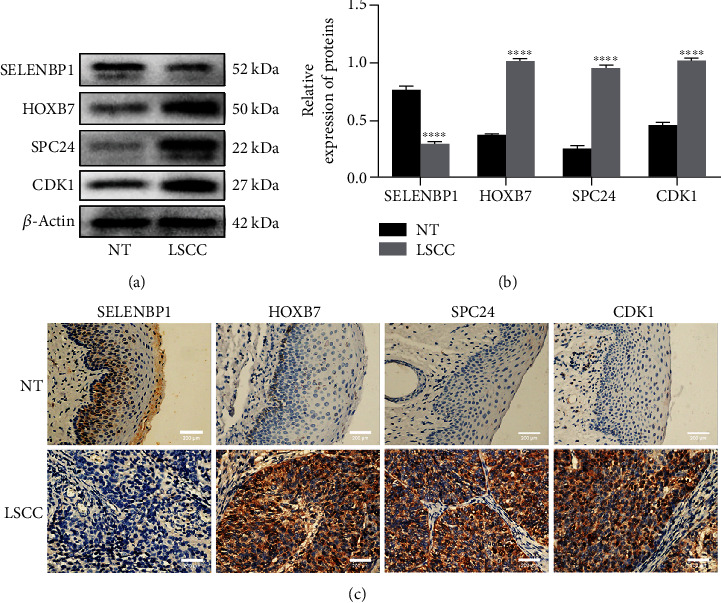
Differences in protein expression of four hub genes. (a) Western blot analysis was performed to quantify the protein expression of SELENBP1, HOXB7, SPC24, and CDK1 in the LSCC and NT groups. (b) Protein levels were normalized to *β*-actin. Quantification of protein levels such as those in (a) is shown in (b). (c) Representative immunostaining of SELENBP1, SPC24, HOXB7, and CDK1 in LSCC and NT groups. All magnification 400x. Each data was presented as mean ± SEM (*n* ≥ 6/group). ^∗∗∗∗^*P* < 0.0001 versus the NT group.

The expression of HOXB7, SPC24, SELENBP1, and CDK1 in LSCC samples and adjacent nonneoplastic samples was assessed by WB analysis.  Figure 8 illustrates that the expression of HOXB7, SPC24, and CDK1 in tumor samples was higher than that in nonneoplastic samples, while the expression of SELENBP1 in tumor samples was lower than that in nonneoplastic samples. The IHC results also confirmed these results.

## 4. Discussion

Bioinformatics methods, such as DEG identification, screening of hub genes based on coexpression networks, and survival analysis, have been extensively used to screen potential biomarkers related to LSCC. For example, Chen et al. [24] combined bioinformatics methods such as DEG identification, pathway enrichment analysis, PPI network construction, survival analysis, and TCGA dataset validation to identify potential biomarkers and analyze their predictions. Li [25] and Zhang et al. [26] combined DEG screening, WGCNA, pathway enrichment analysis, and PPI network construction to identify biomarkers. Although the above studies used a large number of bioinformatics tools to screen biomarkers, they faced the problems of single dataset and small sample size. The inconsistency among DEGs reported in different studies is one of the most important factors underlying differential conclusions and may be due to differences in gene expression being caused by a small sample size from a single dataset or to tumor heterogeneity. To minimize variability, on the basis of using RRA to integrate two datasets and thereby obtain DEGs, GSE59102 and GSE51985 were subjected to WGCNA to identify and merge their key modules. Functional analysis of the merged module showed enrichment in mainly the cell cycle, G1/S transition in the mitotic cell cycle, mitotic nuclear division, sister chromatid cohesion, cell proliferation, and chromatin binding. Based on these results, we hypothesize that cell cycle signaling is the key pathway involved in the development of LSCC, which is consistent with Lu et al.'s conclusion [27].

CDK1 was identified as a core regulatory protein by the PPI network. Cox proportional hazards regression and survival analyses confirmed HOXB7, SELENBP1, and SPC24 to be potential biomarkers of LSCC. To explore the interactions of the hub genes, we constructed a coexpression network, which revealed that SPC24 and CDK1 coexpressions are closely related to the occurrence and development of LSCC. CDK1, a member of the Ser/Thr protein kinase family, plays an important role in G1/S and G2/M phase transitions in the eukaryotic cell cycle, and studies have shown that CDK1 can effectively drive the mammalian cell cycle [28, 29]. SPC24 plays roles in mainly the stable formation of kinetochore-microtubule anchors and correct chromosome separation in mitosis [30–32]. The centromere protein T must recruit the outer SPC24 and SPC25 complexes to stabilize kinetochore-microtubule attachment, which is essential for cell division, and this recruitment process depends on the phosphorylation of centromere protein T by CDK1 [33–35]. In addition, perturbations in cdk2- and cdk1-mediated chromosome stability and some aspects of S phase and G2/M control are pivotal events in tumorigenesis [36–38]. Bednarek et al. [39] showed that the gene expression of CDK1 was consistently increased in LSCC. Several researchers have identified that overexpression of SPC24 promotes tumorigenesis. In some cancers, such as hepatocellular carcinoma [40] and lung cancer [41], the OS time of patients with SPC24 high expression is significantly shorter than that of patients with low expression. This conflicts with our results, which may indicate that SPC24 plays different roles in different types of cancer in humans. Zhang et al. [42] suggested that the OS time of LSCC patients with SPC24 high expression is significantly greater than that of patients with low expression, which was consistent with our results. However, they did not include SPC24 in subsequent analyses due to their greater focus on screening for those genes whose high expression was able to increase the risk of LSCC prognosis. The present study identified SPC24 as a hub gene closely associated with LSCC occurrence and development by WGCNA analysis. Univariate and multivariate Cox analyses were secondarily used to evaluate its ability as a prognostic gene. Finally, functional enrichment analysis and coexpression analysis revealed that CDK1 and SPC24 are coexpressed and involved in LSCC progression and development through the cell cycle pathway. We conclude that spc24 is a gene that may associate with LSCC malignancy and could be identified as independent diagnostic and prognostic biomarker. Until now, the role of CDK1 and SPC24 coexpressions in cancer cells has not been reported. We speculate that CDK1 and SPC24 participate in the occurrence and development of LSCC through the cell cycle signaling pathway. SELENBP1, a member of the selenoprotein family that mediates the transport of selenium in cells [43], may be related to the proliferation, metastasis, invasion, and therapeutic resistance of tumor cells [44, 45]. Studies have shown significant downregulation of SELENBP1 in many types of cancer, including lung cancer [46], gastric cancer [47], prostate cancer [48], and HNSC [49]. The HOXB7 gene, a member of the homeobox gene family, is a transcription factor that regulates the development and differentiation of embryonic cells [50]. HOXB7 plays an important role in multiple processes related to tumor formation and progression, including angiogenesis, proliferation, transformation, invasion, and metastasis [51–53]. Some epidemiological studies have demonstrated that overexpression of HOXB7 is correlated with poor prognosis in breast [54], colorectal [55], and gastric [56] cancer, and upregulation of *HOXB7* mRNA and/or protein expression has been reported in melanoma [57], ovarian cancer [58], and esophageal squamous cell carcinoma [59]. de Barros et al. [13] found that HOXC8, HOXC9, HOXC10, HOXA10, HOXD10, HOXA11-S1, HOXD11, and HOXC13 were significantly overexpressed in LSCC tissues compared with normal tissues. Moreover, HOXC8, HOXD10, and HOXD11 were confirmed to be involved in biological processes related to tumor formation and progression, such as clone formation and cell metastasis [60–62]. Therefore, HOXB7 may be involved in the initiation and progression of LSCC as a complement to the HOX gene family. In addition, ROC curves showed that these four hub genes could be used as biomarkers for LSCC.

We verified the expression of four hub genes in GSE127165. The expression levels of CDK1, SPC24, and HOXB7 were significantly higher in tumor tissues than in normal tissues. However, no data were available for the expression of SELENBP1, which may be related to LSCC heterogeneity. Subsequently, WB and IHC were performed to verify the differential protein expression between LSCC tissues and adjacent nonneoplastic tissues. The protein expression of CDK1, SPC24, and HOXB7 was higher in LSCC samples than in the adjacent nonneoplastic samples, while the protein expression of SELENBP1 was lower in LSCC samples than in the adjacent nonneoplastic samples, which was consistent with the expression changes in the four hub genes observed in LSCC.

In summary, we herein combined comprehensive bioinformatics and molecular biology techniques to identify and verify four potential diagnostic and prognostic biomarkers that are closely associated with LSCC. The results regarding CDK1, HOXB7, and SELENBP1 were consistent with previous findings. However, the differential expression of SPC24 between LSCC and normal tissues has not yet been reported. Our prediction showed that CDK1 and SPC24 coexist in LSCC and may participate in the occurrence and development of LSCC. As for its deeper mechanism of action, although this is not the focus of this paper, it is also the research direction that our research team is very interested in at present. We look forward to using more molecular experiments to explore and prove how CDK1 regulates SPC24 through the cell cycle signal pathway in subsequent studies, thus participating in the occurrence and development of LSCC. The limitations of this study are that most of the results were obtained through the analysis of public data and that geographical or ethnic differences may have existed in the clinical samples obtained from hospitals. Therefore, additional research involving a larger population and elucidation of molecular mechanisms is needed to confirm our findings.

## 5. Conclusions

Our study elucidates pathways and hub genes that are potentially related to LSCC. We herein identify four hub genes, CDK1, SPC24, HOXB7, and SELEBNP1, as candidate diagnostic and prognostic biomarkers of LSCC. Moreover, we propose, for the first time, that SPC24 is a gene that may associate with LSCC malignancy and is a novel therapeutic target. We predict that CDK1 and SPC24 coexpressions are closely associated to the occurrence and progression of LSCC. However, further molecular experiments are needed to clarify their specific mechanisms. These findings may provide new insights into the etiology and prognosis of LSCC.

## Figures and Tables

**Table 1:** Information regarding GSE59102 and GSE51985.

Dataset	Platform	Number of samples (tumors/controls)
GSE59102	GPL6480	29/13
GSE51985	GPL10558	10/10

**Table 2:** Clinicopathological parameters and SPC24 expression according to the TCGA database.

SPC24 mRNA expression					
Parameters	Group	Low (*n* = 56)	High (*n* = 55)	*X* ^2^	*P* value
Age (mean ± SD)		61.61 ± 8.93	62.05 ± 9.67		
Gender	Female	48	43	92.95	0.39
	Male	8	12		
Clinical stage	I/II	7	5	89.46	0.5
	III/IV	47	50		
Living status	Living	19	31	512.31	0.02
	Dead	37	24		

**Table 3:** Univariate and multivariate Cox regression analysis of SPC24 clinical pathologic features according to the TCGA database.

Parameters OS	Univariate analysis				Multivariate analysis			
	HR	Lower_95%	Upper_95%	*P* value	HR	Lower_95%	Upper_95%	*P* value
Age < 60 vs. ≥60	-0.14	0.47	1.6	0.66	0.29	0.15	0.57	0.0004
Gender female vs. male	-1.2	0.15	0.59	0.0005				
Clinical stage I/II vs. III/IV					0.36	0.16	0.78	0.01
Smoking 1 vs. 2	0.33	0.12	0.9	0.03	0.4	0.15	1.12	0.08
Smoking 1 vs. 3	0.15	0.04	0.55	0.005	0.2	0.05	0.78	0.02
Smoking 1 vs. 4	0.31	0.11	0.89	0.029	0.44	0.15	1.29	0.13
*SPC24* expression low vs. high	-0.41	0.44	0.99	0.044	0.33	0.16	0.69	0.003

## Data Availability

All data in this article can be obtained from the author.

## Supplementary Materials

Supplementary Materials Table S1: clinicopathological parameters and HOXB7 expression according to the TCGA database. Table S2: univariate and multivariate Cox regression analysis of HOXB7 clinical pathologic features according to the TCGA database. Table S3: clinicopathological parameters and SELENBP1 expression according to the TCGA database. Table S4: univariate and multivariate Cox regression analysis of SELENBP1 clinical pathologic features according to the TCGA database. Table S5: clinicopathological parameters and CDK1 expression according to the TCGA database. Table S6: univariate and multivariate Cox regression analysis of CDK1 clinical pathologic features according to the TCGA database. Figure S1: (a) functional enrichment analysis of cluster 1. Functional enrichment analysis revealed that the selected genes were related to cell cycle, mitotic nuclear division, and cell division. (b) Functional enrichment analysis of cluster 2. Functional enrichment analysis revealed that the selected genes were related to pathways in cancer, extracellular region, endoplasmic reticulum lumen, and collagen catabolic process. (c) Functional enrichment analysis of cluster 3. Functional enrichment analysis revealed that the selected genes were related to protein binding, ATP binding, herpes simp, hepatitis C, type I interferon signaling pathway, response to virus, and defense response to virus.
