# Supplementary material for: Cell Cycle-Related Gene SPC24: A Novel Potential Diagnostic and Prognostic Biomarker for Laryngeal Squamous Cell Cancer
Source: Biomed Res Int. 2023 Jan 21;2023:1733100. doi: 10.1155/2023/1733100 (PMC9884166; doi:10.1155/2023/1733100)
Supplement: Supplementary Materials — Table S1: clinicopathological parameters and HOXB7 expression according to the TCGA database. Table S2: univariate and multivariate Cox regression analysis of HOXB7 clinical pathologic features according to the TCGA database. Table S3: clinicopathological parameters and SELENBP1 expression according to the TCGA database. Table S4: univariate and multivariate Cox regression analysis of SELENBP1 clinical pathologic features according to the TCGA database. Table S5: clinicopathological parameters and CDK1 expression according to the TCGA database. Table S6: univariate and multivariate Cox regression analysis of CDK1 clinical pathologic features according to the TCGA database. Figure S1: (a) functional enrichment analysis of cluster 1. Functional enrichment analysis revealed that the selected genes were related to cell cycle, mitotic nuclear division, and cell division. (b) Functional enrichment analysis of cluster 2. Functional enrichment analysis revealed that the selected genes were related to pathways in cancer, extracellular region, endoplasmic reticulum lumen, and collagen catabolic process. (c) Functional enrichment analysis of cluster 3. Functional enrichment analysis revealed that the selected genes were related to protein binding, ATP binding, herpes simp, hepatitis C, type I interferon signaling pathway, response to virus, and defense response to virus. [file 1733100.f1.docx]

Table S1: Clinicopathological parameters and HOXB7 expression according to the TCGA database

| **HOXB7** mRNA expression | | | | | |
| --- | --- | --- | --- | --- | --- |
| Parameters | Group | Low(n = 56) | High(n = 55) | X2 | P value |
| Age (Mean ± SD) |  | 61.61+8.93 | 62.05+9.67 |  |  |
| Gender | Female | 48 | 43 | 87.31 | 0.59 |
|  | Male | 8 | 12 |  |  |
| Clinical stage | I/II | 7 | 5 | 479.` | 0.21 |
|  | III/IV | 47 | 50 |  |  |
| Living status | Living | 19 | 31 | 93.5 | 0.41 |
|  | Dead | 37 | 24 |  |  |

Table S2: Univariate and multivariate Cox regression analysis of HOXB7 clinical pathologic features according to the TCGA database

| Parameters OS | Univariate analysis | | | |  | Multivariate analysis | | | |
| --- | --- | --- | --- | --- | --- | --- | --- | --- | --- |
|  | HR | lower_95% | upper_95% | P-value |  | HR | lower_95% | upper_95% | P-value |
| age <60 vs >=60 | -0.14 | 0.47 | 1.6 | 0.66 |  | 0.29 | 0.15 | 0.57 | 0.0004 |
| gender female vs male | -1.2 | 0.15 | 0.59 | 0.0005 |  |  |  |  |  |
| clinical stage I/II vs III/IV |  |  |  |  |  | 0.36 | 0.16 | 0.78 | 0.01 |
| smoking 1 vs 2 | 0.33 | 0.12 | 0.9 | 0.03 |  | 0.4 | 0.15 | 1.12 | 0.08 |
| smoking 1 vs 3 | 0.15 | 0.04 | 0.55 | 0.005 |  | 0.2 | 0.05 | 0.78 | 0.02 |
| smoking 1 vs 4 | 0.31 | 0.11 | 0.89 | 0.029 |  | 0.44 | 0.15 | 1.29 | 0.13 |
| *HOXB7* expression low vs high | 0.44 | 1.1 | 2.2 | 0.01 |  | 1.61 | 1.13 | 2.30 | 0.008 |

Table S3: Clinicopathological parameters and SELENBP1 expression according to the TCGA database

| SELENBP1 mRNA expression | | | | | |
| --- | --- | --- | --- | --- | --- |
| Parameters | Group | Low(n = 56) | High(n = 55) | X2 | P value |
| Age (Mean ± SD) |  | 61.61+8.93 | 62.05+9.67 |  |  |
| Gender | Female | 48 | 43 | 88.43 | 0.56 |
|  | Male | 8 | 12 |  |  |
| Clinical stage | I/II | 7 | 5 | 505.59 | 0.05 |
|  | III/IV | 47 | 50 |  |  |
| Living status | Living | 19 | 31 | 100.23 | 0.24 |
|  | Dead | 37 | 24 |  |  |

Table S4 Univariate and multivariate Cox regression analysis of SELENBP1 clinical pathologic features according to the TCGA database

| Parameters OS | Univariate analysis | | | |  | Multivariate analysis | | | |
| --- | --- | --- | --- | --- | --- | --- | --- | --- | --- |
|  | HR | lower_95% | upper_95% | P-value |  | HR | lower_95% | upper_95% | P-value |
| age <60 vs >=60 | -0.14 | 0.47 | 1.6 | 0.66 |  | 0.29 | 0.15 | 0.57 | 0.0004 |
| gender female vs male | -1.2 | 0.15 | 0.59 | 0.0005 |  |  |  |  |  |
| clinical stage I/II vs III/IV |  |  |  |  |  | 0.36 | 0.16 | 0.78 | 0.01 |
| smoking 1 vs 2 | 0.33 | 0.12 | 0.9 | 0.03 |  | 0.4 | 0.15 | 1.12 | 0.08 |
| smoking 1 vs 3 | 0.15 | 0.04 | 0.55 | 0.005 |  | 0.2 | 0.05 | 0.78 | 0.02 |
| smoking 1 vs 4 | 0.31 | 0.11 | 0.89 | 0.029 |  | 0.44 | 0.15 | 1.29 | 0.13 |
| *SELENBP1* expression low vs high | -0.34 | 0.54 | 0.93 | 0.01 |  | 0.71 | 0.54 | 0.94 | 0.02 |

Table S5: Clinicopathological parameters and CDK1 expression according to the TCGA database

| CDK1 mRNA expression | | | | | |
| --- | --- | --- | --- | --- | --- |
| Parameters | Group | Low(n = 56) | High(n = 55) | X2 | P value |
| Age (Mean ± SD) |  | 61.61+8.93 | 62.05+9.67 |  |  |
| Gender | Female | 48 | 43 | 90.69 | 0.43 |
|  | Male | 8 | 12 |  |  |
| Clinical stage | I/II | 7 | 5 | 488.75 | 0.07 |
|  | III/IV | 47 | 50 |  |  |
| Living status | Living | 19 | 31 | 87.44 | 0.53 |
|  | Dead | 37 | 24 |  |  |

Table S6: Univariate and multivariate Cox regression analysis of CDK1 clinical pathologic features according to the TCGA database

| Parameters OS | Univariate analysis | | | |  | Multivariate analysis | | | |
| --- | --- | --- | --- | --- | --- | --- | --- | --- | --- |
|  | HR | lower_95% | upper_95% | P-value |  | HR | lower_95% | upper_95% | P-value |
| age <60 vs >=60 | -0.14 | 0.47 | 1.6 | 0.66 |  | 0.29 | 0.15 | 0.57 | 0.0004 |
| gender female vs male | -1.2 | 0.15 | 0.59 | 0.0005 |  |  |  |  |  |
| clinical stage I/II vs III/IV |  | 0 | Inf |  |  | 0.36 | 0.16 | 0.78 | 0.01 |
| smoking 1 vs 2 | 0.33 | 0.12 | 0.9 | 0.03 |  | 0.4 | 0.15 | 1.12 | 0.08 |
| smoking 1 vs 3 | 0.15 | 0.04 | 0.55 | 0.005 |  | 0.2 | 0.05 | 0.78 | 0.02 |
| smoking 1 vs 4 | 0.31 | 0.11 | 0.89 | 0.029 |  | 0.44 | 0.15 | 1.29 | 0.13 |
| *CDK1* expression low vs high | 0.03 | 0.67 | 1.6 | 0.89 |  | 1.16 | 0.77 | 1.77 | 0.47 |


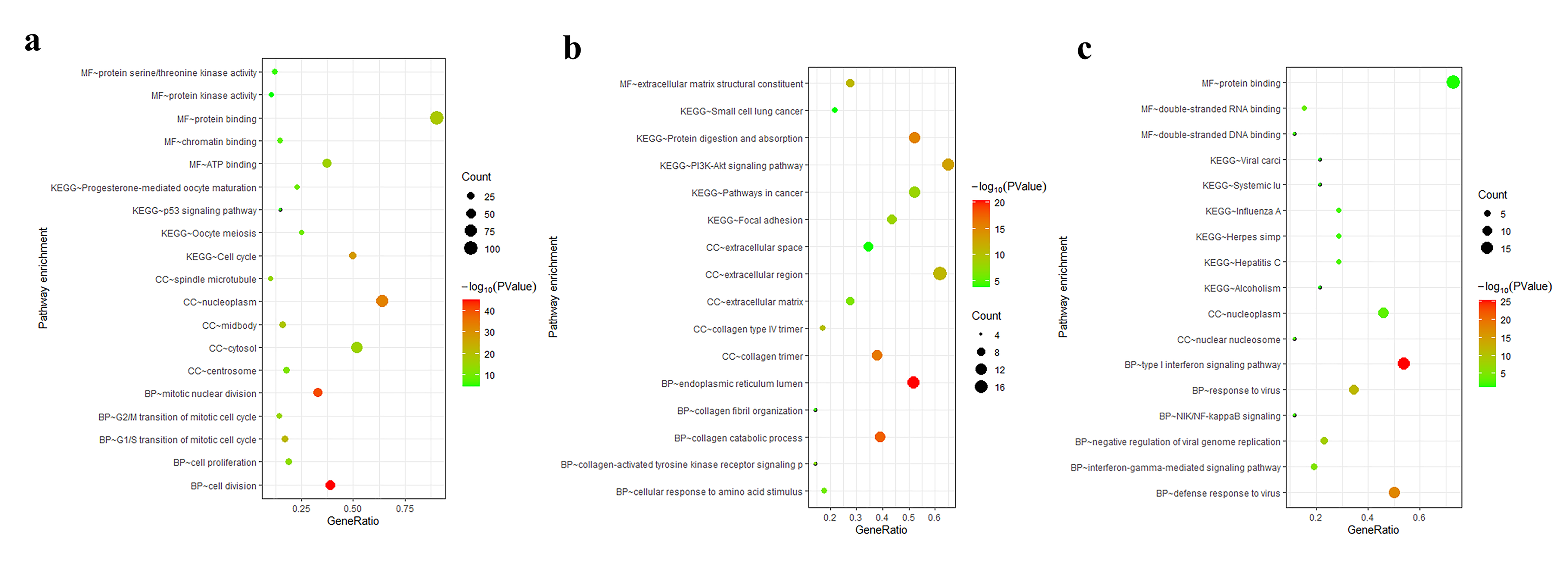


Figure S1:（a）Functional enrichment analysis of cluster 1. Functional enrichment analysis revealed that the selected genes were related to cell cycle, mitotic nuclear division and cell division. (b)Functional enrichment analysis of cluster 2. Functional enrichment analysis revealed that the selected genes were related to pathways in cancer,extracellular region, endoplasmic reticulum lumen and collagen catabolic process. (c)Functional enrichment analysis of cluster 3. Functional enrichment analysis revealed that the selected genes were related to protein binding, ATP binding, Herpes simp, Hepatitis C, type I interferon signaling pathway, response to virus, defense response to virus.
